# Moving microcapillary antibiotic susceptibility testing (mcAST) towards the clinic: unravelling kinetics of detection of uropathogenic *E. coli*, mass-manufacturing and usability for detection of urinary tract infections in human urine†

**DOI:** 10.1039/d2sd00138a

**Published:** 2023-05-03

**Authors:** Sarah H. Needs, Jeremy Pivetal, Jessica Hayward, Stephen P. Kidd, HoYin Lam, Tai Diep, Kiran Gill, Martin Woodward, Nuno M. Reis, Alexander D. Edwards

**Affiliations:** a Reading School of Pharmacy, University of Reading Whiteknights Campus Reading RG6 6AD UK s.h.needs@Reading.ac.uk a.d.edwards@reading.ac.uk +44(0)7906014116 +44(0)118 378 4253; b Hampshire Hospitals NHS Foundation Trust Basingstoke and North Hampshire Hospital Basingstoke RG24 9NA UK; c Department of Food and Nutrition Sciences, University of Reading Whiteknights Campus Reading RG6 6DX UK; d Department of Chemical Engineering and Centre for Biosensors, Biodevices and Bioelectronics (C3Bio), University of Bath Claverton Down Bath BA2 7AY UK n.m.reis@bath.ac.uk +44(0)1225 383 369; e Capillary Film Technology (CFT) Daux Road Billingshurst RH14 9SJ UK

## Abstract

Innovation in infection based point-of-care (PoC) diagnostics is vital to avoid unnecessary use of antibiotics and the development of antimicrobial resistance. Several groups including our research team have in recent years successfully miniaturised phenotypic antibiotic susceptibility tests (AST) of isolated bacterial strains, providing validation that miniaturised AST can match conventional microbiological methods. Some studies have also shown the feasibility of direct testing (without isolation or purification), specifically for urinary tract infections, paving the way for direct microfluidic AST systems at PoC. As rate of bacteria growth is intrinsically linked to the temperature of incubation, transferring miniaturised AST nearer the patient requires building new capabilities in terms of temperature control at PoC, furthermore widespread clinical use will require mass-manufacturing of microfluidic test strips and direct testing of urine samples. This study shows for the first-time application of microcapillary antibiotic susceptibility testing (mcAST) directly from clinical samples, using minimal equipment and simple liquid handling, and with kinetics of growth recorded using a smartphone camera. A complete PoC–mcAST system was presented and tested using 12 clinical samples sent to a clinical laboratory for microbiological analysis. The test showed 100% accuracy for determining bacteria in urine above the clinical threshold (5 out of 12 positive) and achieved 95% categorical agreement for 5 positive urines tested with 4 antibiotics (nitrofurantoin, ciprofloxacin, trimethoprim and cephalexin) within 6 h compared to the reference standard overnight AST method. A kinetic model is presented for metabolization of resazurin, demonstrating kinetics of degradation of resazurin in microcapillaries follow those observed for a microtiter plate, with time for AST dependent on the initial CFU ml^−1^ of uropathogenic bacteria in the urine sample. In addition, we show for the first time that use of air-drying for mass-manufacturing and deposition of AST reagents within the inner surface of mcAST strips matches results obtained with standard AST methods. These results take mcAST a step closer to clinical application, for example as PoC support for antibiotic prescription decisions within a day.

## Abstract 2

Point-of-care antibiotic susceptibility test for urinary tract infections.

## Graphical abstract

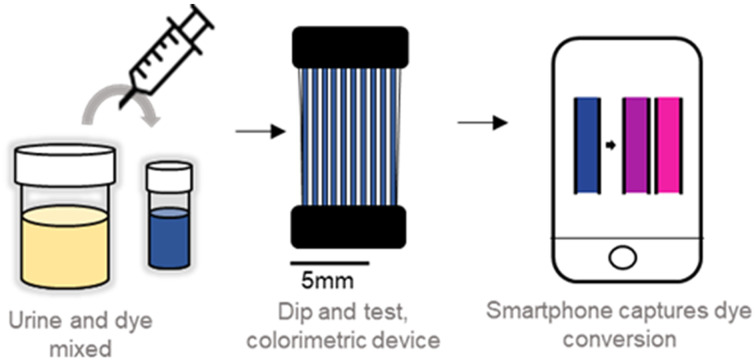

## Introduction

Antimicrobial resistance (AMR) is a global challenge to human health. The WHO recommends improving diagnostics, and national government strategies to combat AMR also prioritise innovation in diagnostics.^1,2^ Point of care (PoC) and point of need devices that perform microbiological tests, such as antimicrobial susceptibility tests (AST), are urgently needed. AST methods can be phenotypic measurements or genotypic nucleic acid based. While genotypic measurements (molecular methods such as MS/MS and PCR detection) have the potential to provide rapid results (<30 minutes) and provide important information such as identification, as well as detect known resistance mechanisms, they do not always map directly onto phenotypic susceptibility. Culture (phenotypic) methods therefore remain the current clinical gold standard methods for determining AST. These include disc diffusion (DD) and broth microdilution (BMD), and depend on functional measurement of growth of pure bacterial cultures in the presence of antimicrobials. These tests can only be performed in qualified *in vitro* diagnostic microbiology laboratories and require 48–72 h for a result. This long time to result means that a patient presenting with suspected infection are often prescribed antibiotics empirically before laboratory results are returned for diagnosis. Therefore, there is a major need for rapid phenotypic AST to reduce this period of diagnostic uncertainty and/or move the testing closer to the patient, without the infrastructures that can be found in microbiology laboratories.

Microfluidic systems offer many benefits that fit the requirements for PoC testing, including faster time to results, portability and automation; the use of microfluidics for PoC AST has been reviewed.^3,4^ Multiple studies have demonstrated the potential to miniaturise AST into microfluidic systems^5–7^ and in single cell systems.^8–10^ Single cell microscopy or individual particle tracking systems have reported the fastest phenotypic AST results, some in as little as 30 minutes^11–13^ but most report results within 1–3 h.^14–17^ Other methods to measure bacterial activity, including impedance, can also be used to categorise effects of antibiotics in 0.5–1 h.^18,19^ However, there is often a trade-off between accuracy, accessibility and affordability. For example, rapid microscopy methods may require more expensive and bulkier optics compared to simpler growth readouts, and may be better suited to laboratory or centralised testing sites. Many microfluidic designs can also pose a challenge for scalable, affordable manufacture. The architecture of many prototype proof-of-concept devices are complex, and the deposition of liquid and dried reagents can be challenging for mass-manufacture.^20^ Paper diagnostics can offer more affordable microfluidics and are typically made of low-cost materials. Paper based PoC tests have used colorimetric systems such as resazurin or chromogenic media to detect bacterial growth in the presence of different antibiotics.^21,22^ However, compartments in paper-based systems are more difficult to keep confined using paper systems and depending on design, antibiotics can diffuse out of individual compartments changing the concentration in the system.

Urinary tract infections (UTIs) are an extremely common infection and are the second most common reason for antibiotic prescriptions^23^ with a high rate of inappropriate antibiotic use.^24^ Currently, the only PoC test for UTI is a simple, low-cost dipstick that can measure indirect markers of infection such as nitrites and pH and take seconds to perform, but cannot indicate antimicrobial resistance. Standard of care (SoC) laboratory testing can require two overnight culture steps, firstly streaking on agar plates to generate a pure monoculture of the pathogen, followed by second overnight AST; if the first step is followed by a slow identification stage (*e.g.* without access to MS/MS) the process can take longer than 2 days. Rapid AST methods are emerging that can shorten the time for isolate testing.^25,26^ Direct testing of urine for AST is a potential strategy for the development of significantly faster tests by avoiding overnight culture for isolation, and more suitable for PoC use by avoiding complex manipulations such as picking individual colonies.

Direct AST has been demonstrated from positive blood cultures and is used in many clinical settings.^25,27^ Direct disc diffusion (dDD) studies have shown accurate results from directly tested samples^28^ reducing time to result. Other methods such as premade agar plates with antibiotic containing sections have also been found to be accurate used directly from urine samples.^29,30^ However, standard protocols for AST still require 16–20 h incubation before determining the results. Shorter incubation timepoints have been used for dDD for infections in which time to result is crucial, such as sepsis, which uses timepoints as rapid as 4 h to measure susceptibility with disc diffusion plates.^31^ The time to result will depend on the plating density and doubling time of the bacteria in the system, which will affect the accuracy of the AST, but the inoculum effect is less pronounced for disc diffusion which is why direct testing using this method can be used. Even within the recommended inoculum density for broth microdilution, variation in MIC can be seen.^32^ Insight into the kinetics of growth in liquid media over a range of inoculum densities in microcapillaries is therefore vital to determine when tests can be read.

Several microfluidic^5,6,8,33–35^ and other novel methods^36–38^ have tested the feasibility of direct urine AST in liquid and even reporting functional AST in under 3 h.^5,36,37,39,40^ While some systems have reported rapid time to result, this must be balanced with specific use cases. In many cases the point at which a urine samples is taken is not where it will be tested and therefore needs transportation to a diagnostic lab. Devices need to be designed taking into account the environment it will enter, and following careful trade-offs between ideal test criteria, outlined by the REASSURED (real-time connectivity, ease of specimen collection, affordable, sensitive, specific, user-friendly, rapid, equipment-free, and delivered) guidance for PoC devices.^41^ For example, some systems require extra sample handling to exchange the urine with growth media to minimise matrix interference.^6,40^

We previously reported a ‘lab-on-a-stick’ that permits functional microbiology tests including the determination of minimum inhibitory concentration (MIC) of antibiotics used in the treatment of UTI.^42,43^ We demonstrated accurate quantification of viable cells in individual microcapillary film strips down to 1 CFU per capillary,^44,45^ and shown that AST in the presence of different urine samples can show significant degree of variation depending on individual differences such as pH and sample dilution.^35^ However, these systems focused on laboratory use requiring laboratory equipment including pipettes, microtitre plates and incubators.

Here we present a complete application of a microcapillary ‘dip and test’ resazurin-based AST system for optically measuring antibiotic susceptibility directly from urine within 6 h. A 3D printed prototype PoC–mcAST device enabled the control of incubation temperature combined with simple optical imaging of colorimetric growth with a smartphone camera. The simplified liquid handling of the test allows it to be performed near patient or outside a laboratory, potentially useful for low-resource settings. Furthermore, we show that two methods of antibiotic deposition within the microcapillary devices can be used, avoiding the need for freeze-drying and thereby ensuring the microcapillary based test devices are compatible with future mass-manufacture.

## Experimental section

### Urine sample collection

Samples were collected in Brand™ Urine beakers under instruction to collect a midstream urine sample. Samples were collected and refrigerated within 4 h of collection. To determine precipitation of resazurin dye, unfiltered urine was tested within 4 h using Uritest 10V Urinalysis strips and Quantofix Ascorbic Acid test (Merck, UK) and pH was determined by pH electrode. Following this urine samples were stored at −20 °C until use. Ethical consent for the collection of urine from healthy donors was received from the University of Reading, reference code 19/59. Informed written consent was obtained from all participants.

Diagnostic remnant clinical samples were collected from Basingstoke and North Hampshire Hospital. Urine samples submitted for microbiological analysis were stored in boric acid containers. Samples were aliquoted (1 mL) into a sterile tube and tested within 48 h of the sample arriving at the hospital laboratory. Urine samples were streaked on UTI ChromoSelect Agar (Merck, UK) following manufacturer's instructions, allowing presumptive identification. The collection of diagnostic remnants from Basingstoke and North Hampshire Hospital was approved by the Health Research Authority (HRA) (IRAS: 316558) and the University of Reading, reference code 22/33.

### Bacterial isolates

*E. coli* ATTC 25922 (LGC Logistics, UK) was used as the quality control strain. Uropathogenic *E. coli* isolates 181717 and 181767 were kindly donated by Frimley Park Hospital, UK. Uropathogenic *E. coli* isolates 2151–2170 were collected at a tertiary care hospital of Pakistan from community acquired UTI patients^46,47^ under a study that was approved by the Ethical Review Board (ERB) of the Pakistan Institute of Medical Sciences. UPEC were identified using standard microbiological and biochemical tests as described before.^48^

### Freeze-dried production of mcAST strips

The fluorinated ethylene propylene microcapillary film (MCF) was manufactured by melt-extrusion by Lamina Dielectrics Ltd (Billingshurst, West Sussex, UK) from a highly transparent fluorinated ethylene propylene co-polymer (FEP-Teflon®) and comprises a ribbon containing an array of 10 capillaries along its length with an average diameter of 270 μm. For each batch, 1–5 m MCF lengths were internally coated by incubation with a 5 mg mL^−1^ solution of PVOH in water (MW 146 000–186 000, >99% hydrolysed, Sigma-Aldrich, UK) at room temperature for a minimum of 2 h.^49^ Coated strips were washed with 5 ml of PBS with 0.5% Tween 20 (Sigma-Aldrich, UK) to remove residual PVOH, and dried by attached one end of the film to a vacuum manifold and air dried for 20 minutes.

To load antibiotics into the capillaries of the PVOH modified MCF, individual microcapillaries of lengths of MCF up to 5 m long were filled using a 30G needle with freshly prepared filter sterilized antibiotic solutions detailed in Table S-1.† Individual test strips were cut to 17 mm lengths, frozen for at least 1 h at −80 °C and freeze-dried using an Edwards Modulyo shelf freeze-drier. Test strips were vacuum sealed and stored at −20 °C for a maximum of a week before use.

### Air-dried production of mcAST strips

MCF was modified with PVOH as described above. To load antibiotics onto the internal surface of the PVOH modified MCF, individual microcapillaries of a length of PVOH modified MCF (up to 5 m) were filled using a 30G needle with freshly prepared filter sterilized antibiotic solutions of gentamicin, tetracycline, trimethoprim, ciprofloxacin, ampicillin, amoxicillin and cefotaxime (Sigma Aldrich, UK) at the following concentrations and solvents: gentamicin 5 mg mL^−1^ in ultrapure water; tetracycline 5 mg mL^−1^ in water; trimethoprim 3 mg mL^−1^ in DMF; ciprofloxacin 3 mg mL^−1^ in 1% HCl; ampicillin 10 mg mL^−1^ in water; amoxicillin 15 mg mL^−1^ water; and cefotaxime 4.8 mg mL^−1^ in water. After 5 minutes of incubation, the excess solution was removed by attaching MCF lengths to a vacuum manifold and dried for 20 minutes per 1 m length, leaving behind a thin film of antibiotic as described previously.^42^ All antibiotics were loaded at concentrations that would lead to release of the breakpoint concentration into the sample (Table S-1†) based on previously determined loading efficiency quantified by LC-MS and confirmed by phenotypic AST experiments using this method.

### Simulated fresh urine and diagnostic remnant urine microcapillary assays

Bacteria were cultured routinely on LB agar and Mueller-Hinton broth (MHB) at 37 °C. Bacteria were diluted to 0.5 McFarland corresponding to approximately 1 × 10^8^ CFU mL^−1^ and then further diluted to the indicated densities into sterile normal human urine samples to produce spiked simulated UTI samples. Resazurin was added to a final concentration of 0.25 mg mL^−1^ resazurin for colorimetric detection. Mueller-Hinton broth was combined with urine such that each concentration of urine contained the equivalent nutrients of a 1× solution, regardless of the urine dilution factor. Samples were tested by dipping test strips in the urine/media/resazurin sample for a few seconds until all capillaries had filled and the sample had reached the top of the device. After sealing the ends by adding a simple end cap, the hot bed was set to 35 °C and maintained within ±2 °C in the portable incubator. Endpoint images were also taken with an Apple iPhone 6s on the diffuse white light background. UPEC susceptibility categorisation results were compared to the MIC determined here.^43^ Details of traditional AST methods can be found in the ESI† including microplate broth microdilution and disc diffusion (S-1).

Diagnostic urine samples were collected in bacteriostatic boric acid containers to be processed for routine clinical analysis. Diagnostic remnant samples were collected in 1 mL aliquots to be tested in the microcapillary test. Because diagnostic remnant samples contained the bacteriostatic boric acid, the samples were diluted 1 : 100 in MHB with a final concentration of 012 mg mL^−1^ resazurin. At lower dilutions, boric acid inhibited bacterial growth. To determine the bacterial load in the diagnostic remnant samples, urine was serially diluted in MHB and spotted (5 μL) onto plate count agar. Agar plates were incubated overnight at 37 °C. Total colonies were counted regardless of colony morphology.

### Data analysis

Images were analysed in ImageJ as previously described.^42,50^ Briefly, images were split into RGB and the absorbance for each capillary was analysed in the red channel. When images were analysed, the largest intensity change was a reduction in red light absorbance, reflecting a major peak absorbance shift to shorter wavelengths following metabolic conversion of resazurin to resorufin. This results in a drop in red channel absorbance detected by the RGB sensor, and accounts for the colour change from blue to pink. Time to resazurin conversion was calculated when signal fell below half the starting absorbance. Endpoint images taken using smartphone camera were scored by eye as growth or no growth based on colour change.

### Modelling cell growth kinetics

We developed a simple kinetic model demonstrating bacteria growth and rate of metabolization of resazurin in microcapillaries to compare with growth observed for a microtiter plate AST. In brief, we considered resazurin metabolization to be an enzymatic process linked to the cell density or CFU ml^−1^. This is in line with *e.g.* the work of ref. 51 that showed the rate of resazurin metabolization is proportional to the biomass concentration. It is therefore possible to establish a correlation between the degradation or conversion *X* of resazurin, the growth rate of bacteria, and the initial cell density CFU ml^−1^. The model involved best fitting a single arbitrary kinetic constant *k*_c_, being the number of moles of resazurin converted per unit of time per CFU. The kinetic model is fully described in the ESI† document.

## Results and discussion

### Direct, PoC–mcAST concept

**Fig. 1:**
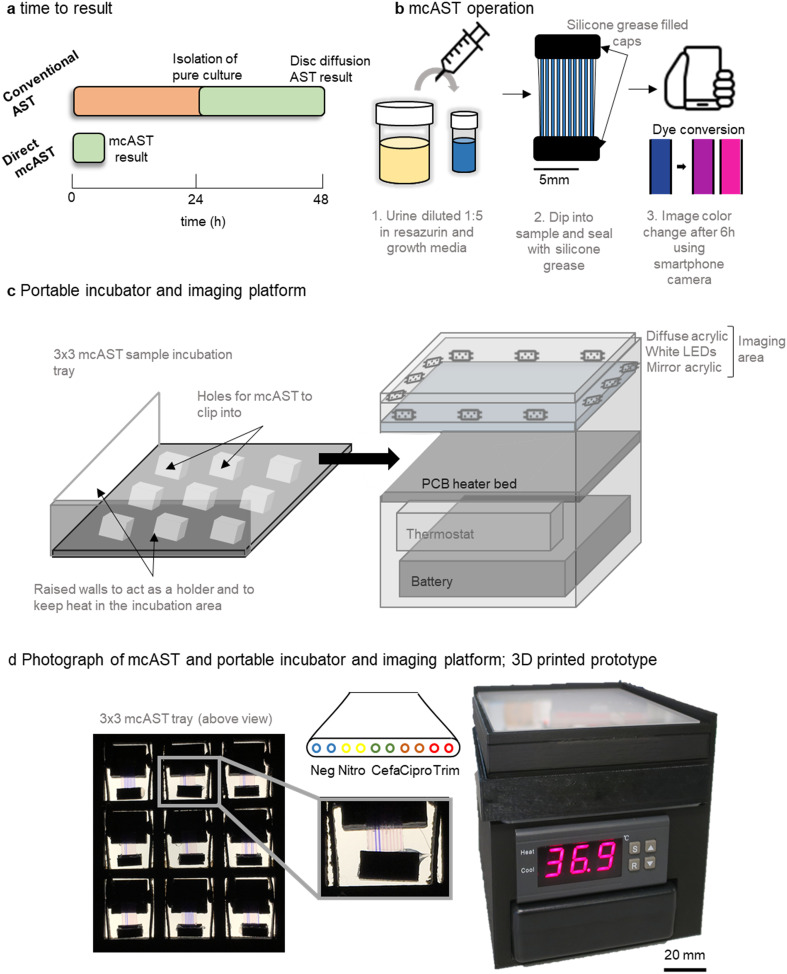
Design and operation of 3D printed prototype PoC–mcAST device. (a) The mcAST systems reports results directly from urine in 6 h. (b) mcAST is operated by diluting urine 1 : 5 in growth media with resazurin. The test strip is dipped into the sample and capillaries fill by capillary action. End caps filled with silicone grease cover each end to prevent sample evaporation. After 6 h incubation the result can be captured digitally using a smartphone camera. Diagram (c) and photograph (d) of the portable system that contains an area for sample incubation and separate imaging surface for digital recording of results. Individual microfluidic dip and test strips are held in a 3 × 3 grid which fits into the incubation stage. After incubation, the 3 × 3 sample grid is removed from the incubation stage and placed on top of the diffuse white backlight and imaged using an iPhone 6s smartphone camera. Neg; no antibiotic growth control, Nitro; nitrofurantoin 64 mg L^−1^, Cefa; cefalexin 16 mg L^−1^, Cipro; ciprofloxacin 0.25 mg L^−1^ and Trim; trimethoprim 4 mg L^−1^.

Current AST methods streak a urine sample on agar, allowing identification, total bacterial count, and isolation of a pure bacterial cultures before AST (Fig. 1a). Direct testing can reduce time to result by ∼24 h by removing the culture step. Here, we show feasibility for a direct urine AST system using the microcapillary AST concept described previously^35,42,43^ that can be performed outside of a diagnostic microbiology laboratory using a portable incubator and smartphone detection.

The proposed workflow of the UTI mcAST uses simple sample loading using capillary rise and minimal liquid handling. The urine sample is diluted 1 : 5 in growth detection media. Each sample was mixed by inversion and a mcAST test strip dipped into each diluted sample. The sample is drawn up by capillary action and the device is sealed by caps filled with silicone grease (Fig. 1b). The mcAST strips consist of 17 mm lengths of microcapillary film, each containing 10 parallel ∼300 μm internal diameter microcapillaries, allowing multiplexing of up to 9 antibiotics plus a growth control (without antibiotic) for each test strip. For this study, duplicate pairs of capillaries contained four freeze dried antibiotics at the breakpoint concentrations. Bacterial growth can be detected using the metabolic indicator, resazurin, and follows the colour change from blue to pink which indicates growth.^43^ Up to 9 mcAST test strips fit in a 3 × 3 frame holder that is incubated at 37 °C in a portable, battery-powered heating chamber, and a smartphone camera captures colour change of the resazurin from blue to pink, with the rack of test strips illuminated with a white LED panel (Fig. 1c and d). The PoC–mcAST device permits rapid repeat dip-testing with multiple test strips, making this approach feasible when more antibiotics or full MICs are needed for a single sample.^43^

An unaddressed challenge required for bringing mcAST out of the lab towards the clinic involves the temperature control and imaging of mcAST strips at the PoC, without dedicated equipment available in a microbiology lab. We therefore developed a portable test reader and incubator. The system has a small footprint, 100 mm × 100 mm, consisting of incubation stage and white diffuse imaging area for recording results. A battery and temperature controller fit underneath, making the system fully portable. Further details of the portable incubator hardware can be found in supplementary methods described in ESI.† Increasingly, more designs for portable and affordable incubation for field microbiology have been developed.^52–54^ Since the mcAST devices are low profile they can be effectively heated from below, without needing a large incubator volume. The incubation stage was prototyped using a heated bed designed for 3D printers, comprising a simple resistive heating element on a printed circuit board (PCB). This hotbed stays within ±2 degrees of the set temperature (Fig. S-1†), controlled by a low-cost temperature controller installed underneath. A portable battery pack in the form of a consumer uninterruptible power supply (UPS) unit could easily run the system for at least 6 h. Individual mcAST devices clip into a 3 × 3 holder that slides on top of the hotbed and can be removed and placed on top of the imaging area (Fig. 1c).

Different sensors have been incorporated into microfluidic systems including colorimetric/fluorescence indicators or electrical signals.^33^ Resazurin has been incorporated in a number of traditional microbiological AST methods^55^ as well as microfluidic devices.^6–8,22,56–64^ Resazurin is an attractive sensor as it can be used both colourimetrically or fluorescently, and it measures growth of most organisms without requiring specific microbial enzymes to be present.^65^ Conversion of resazurin to resorufin is non-reversible, however, the fluorescent resorufin can be reduced further to a non-fluorescent and colourless, dihydroresorufin which is reversible. For this reason, colorimetric detection was chosen to maintain a stable endpoint image. For the accurate digitisation of the results, a diffuse white light platform was built. The imaging area contains a strip of white LEDs facing into the imaging area with a piece of diffuse opal acrylic above. A piece of mirrored acrylic is placed underneath to make the LED illumination more even throughout the imaging area (Fig. 1c). When an image is taken, the 3 × 3 mcAST tray is removed from the incubation area and placed onto the imaging area. The results can be captured using a smartphone camera.

### Reproducible growth detection across wide dynamic range of starting cell density

**Fig. 2:**
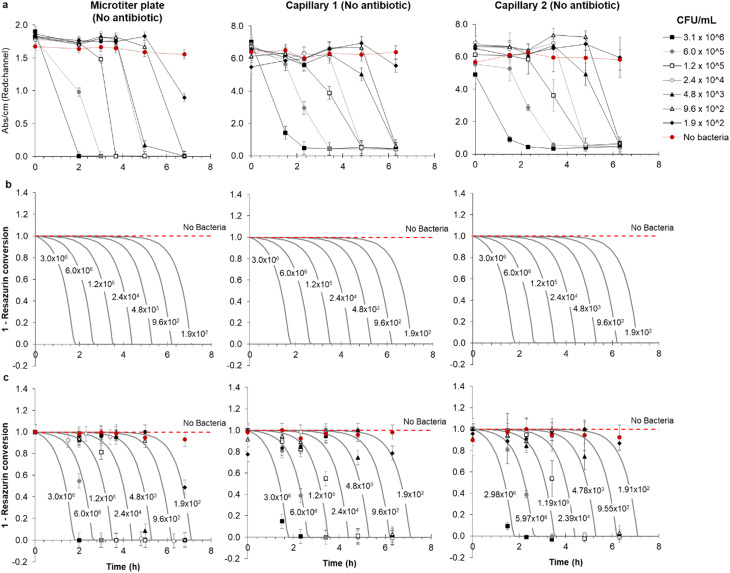
Comparison of detection kinetics from simulated clinical urine samples between microplate and replicate microcapillaries at clinically relevant range of starting pathogen density. (a) Clinical isolate 181717 was diluted into simulated urine and tested in three replicate microcapillary test strips loaded in duplicate with no antibiotic in capillary 1 and 2 and incubated alongside a replicate microtitre plate wells. Plates and test strips were imaged at the indicated times, and red absorbance measured to indicate bacterial growth. (b) Modelling of metabolization (conversion) of resazurin in the microtiter plate and two representative microcapillary. (c) Combined experimental and modelled conversion of resazurin, showing the quality of fit. This is further demonstrated by Fig. S-3,† showing the same kinetic model with the same kinetic parameters can reasonably predict resazurin conversion in both the microtiter plate and microcapillaries. In all figures, error bars represent ±1 SD from mean absorbance of the three replica samples.

Functional AST inherently requires detection of bacterial growth, therefore a detailed understanding of growth kinetics of bacterial cells in microfluidic devices is essential. It is critical to understand if growth in microfluidic devices occurs as rapidly as in conventional culture and if cell density affects the test result over the clinically important range. To explore the feasibility of direct urine testing and study the impact of variable pathogen density, a panel of simulated patient samples was prepared in microtitre plates comprising synthetic urine spiked with 5-fold dilutions of a representative *E. coli* uropathogenic clinical isolate (Fig. 2). Colorimetric growth detection was recorded at regular intervals over 6 h incubation in both microwells and mcAST device. As expected, blue to pink transition was fastest at high cell densities (Fig. 2). The drop in red absorbance was highly reproducible, with duplicate capillaries in triplicate test strips showing highly consistent kinetics of colour change (Fig. 2 and S-2†). Bacteria are distributed in the microcapillaries based on Poisson distribution.^44^ Based on this, the limit of detection (LOD) in a 1 microlitre capillary volume device is approximately 5 × 10^3^ CFU mL^−1^, in which 99% of capillaries all contain at least 1 CFU. The latest growth detection timepoint at this density is expected around 6 h for *E. coli* (Fig. 2). The LOD can be adjusted by simply changing the length and therefore the inner volume of the microcapillary for the test. By modelling the kinetics of degradation or conversion, *X* of resazurin, intrinsically linked to the accumulation of *E. coli* cells, it was observed that growth of bacteria in microcapillaries was remarkably similar to that in the microtiter plate (Fig. 2), which is not surprising as the soluble metabolic dye conversion is not diffusion-limited, and therefore one of the major speed benefits of assay miniaturisation for some bioassays – reduced diffusion time – is not expected to play a major role for this mode of bacterial growth detection.

A rate of cell growth, *μ* = 1.8 h^−1^ required for the kinetic model was estimated from the initial experimental data (Fig. 2a) for microcapillaries and microwells by determining the mean increase in time to detectable colour change for each 5-fold dilution of starting cell density, measured as 0.92 h and 0.94 h respectively, which corresponds to a doubling time of ∼23 minutes. This is in line with the maximum growth rates and doubling times widely reported in literature for *E. coli* at 37 °C. In brief, the kinetic model considered the rate of degradation of resazurin follows a first order enzymatic conversion which is linked to the cell density and therefore cell growth. We have considered increase in cell density followed a typical exponential growth, which is realistic considering the modest initial CFU ml^−1^, in the range of 1.9 × 10^2^–3.1 × 10^6^ CFU ml^−1^, the abundance of carbon source in the growing media, and the short time scale of the experiments.

Figures show the rate of degradation or conversion of resazurin can be predicted using exactly the same growth model which used a single, arbitrary, kinetic parameter, *k*_c_ being the number of moles of resazurin converted per CFU per unit of time. Taking into consideration the experimental data shown for the microwell plate and microcapillaries shown in Fig. 2a, we have estimated *k*_c_ = 2.5 × 10^−8^ μmol h^−1^ CFU^−1^ using best-fitting based on minimum square differences. The model was shown to reasonably predict the experimental data, as shown in Fig. S-3† and helped highlighting two important aspects: firstly, replicate microcapillaries match tightly, and secondly bacteria growth in microcapillaries follows the same kinetics as conventional ‘macro’ fluidic systems, confirming that measurement of bacterial growth in ‘tiny’ volumes is equivalent to microwells.

### Direct mcAST in human urine spiked with clinical isolates diluted 1 : 5

To validate the mcAST system for direct AST, 20 uropathogenic *E. coli* (UPEC) isolates, and *E. coli* 25922 quality control strain were spiked into real human urine samples. Resazurin based broth microdilution in microtitre plates was used as a reference test to determine MIC and categorise susceptibility and resistance for each isolate (Dataset S1). Four antibiotics used for the treatment of UTIs were used at a single breakpoint concentration based on EUCAST clinical breakpoint tables for *Enterobacterales* (Table S-1†). Nitrofurantoin, ciprofloxacin, cefalexin and trimethoprim were loaded into duplicate capillaries. Two capillaries were left negative for the growth control. The results were designated susceptible if resazurin remained blue in the presence of the antibiotic to the breakpoint concentration or resistant if the capillaries changed colour to pink/colourless and an image was taken after 6 h using a smartphone camera.

To simulate the effect of direct testing, two inoculum densities were used and spiked into two separate urine samples. UPEC isolates were spiked into urine at the threshold density for UTI diagnosis, 10^5^ CFU mL^−1^, simulating the lowest concentration of bacteria in a urine flagged as culture positive, and therefore the longest to result. A higher density was chosen based on literature, which reports a wide variation of bacterial density in urine samples affected by different patient group or type of infection, but following one study of paediatric positive urine samples that found a mean colony count of 1.7 × 10^7^ CFU mL^−1^,^66^ we chose 10^7^ CFU mL^−1^ as a simulated higher load infection.

Previously we reported that some components of urine can affect AST results, however, dilution by 1 : 5 minimised many of these effects^35^ including speed of growth detection^44^ (Table S-2†). After a 1 : 5 dilution of urine in indicator media, the bacteria density tested was 2 × 10^4^ CFU mL^−1^ and 2 × 10^6^ CFU mL^−1^ representing the threshold and high infection load samples respectively. Nitrofurantoin, cefalexin and trimethoprim showed 100% categorical agreement with reference method microplate BMD at both bacterial cell densities after 6 h incubation at 37 °C (Table 1). Ciprofloxacin showed 90–93% categorical agreement at the different cell densities. For all the major errors identified for ciprofloxacin the MIC by BMD was 0.25 mg L^−1^, the same as the breakpoint concentration. At this concentration it is very likely to see differences in R/S categorisation following standard AST techniques.

**Table 1:** Concordance of mcAST for four antibiotics used for UTI treatment using 20 UPEC isolates, and QC strain spiked in 2 individual urine samples at 6 h incubation, representing 42 simulated UTI urines

Inoculum density		10^7^ CFU mL^−1^			10^5^ CFU mL^−1^		
Antibiotic	Prevalence of resistance%^a^	Concordance%	False resistance%	False susceptibility%	Concordance%	False resistance%	False susceptibility%
Nitrofurantoin	(0/21) 0%	100% (42/42)	—	—	100% (42/42)	—	—
Cefalexin	(19/21) 90%	100% (42/42)	—	—	100% (42/42)	—	—
Trimethoprim	(18/21) 86%	100% (42/42)	—	—	100% (42/42)	—	—
Ciprofloxacin	(18/21) 86%	93% (39/42)	5% (2/42)	2% (1/42)	90% (38/42)	2% (1/42)	7% (3/42)

a As determined by BMD.

It is worth noting that the isolates tested here have a high prevalence of resistance for cefalexin, trimethoprim and ciprofloxacin, making identification of false resistance not possible and the inverse for nitrofurantoin. Furthermore, the majority of isolates had MIC values that are multiple dilutions below the breakpoint concentration, ranging from ≤2–16 mg L^−1^ with a EUCAST breakpoint of 64 mg L^−1^, so even if the MIC was affected by the urine matrix, this might not result in a change in susceptibility when tested at this breakpoint.

**Fig. 3:**
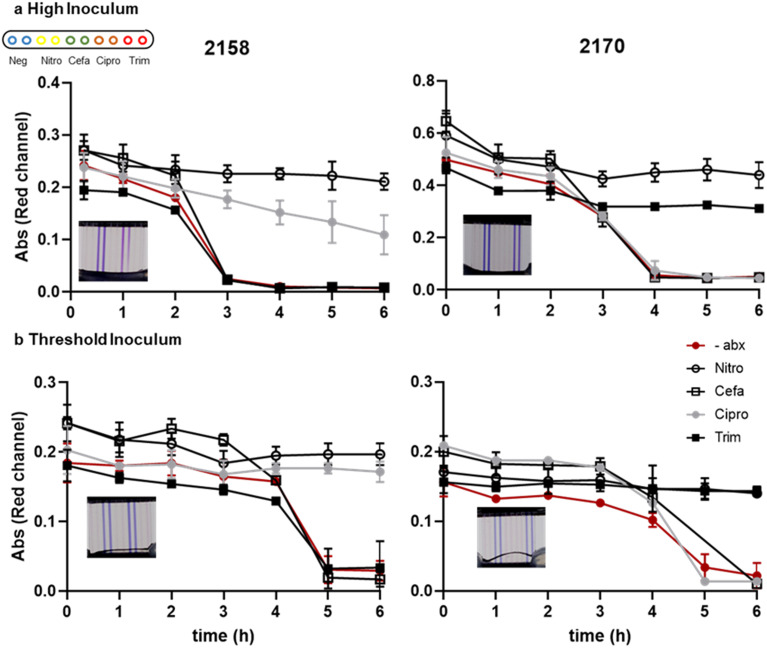
Diagnostic threshold inoculum detected within 6 h. Growth kinetics of UPEC isolates 2158 and 2170 spiked into urine at 3 × 10^7^ and 10^5^ CFU mL^−1^ and diluted 1 : 5 in Mueller-Hinton broth with resazurin. Data indicates mean ± SD of duplicate capillaries. Image insert represents the 6 hour endpoint image.

We measured the kinetics of growth detection and AST measurement for two clinical isolates, 2158 and 2170, spiked into urine at the two different cell densities. The recommended inoculum density to perform AST is 5 × 10^5^ CFU mL^−1^. The inoculum effect (IE) is a well characterised phenomena by an increase in the observed MIC when tested with a higher density of bacteria. In samples with unknown bacterial densities it is therefore important that the test device can operate over a high dynamic range of bacteria from typical diagnostic threshold of 10^5^ CFU mL^−1^ to above 10^7^ CFU mL^−1^.^67^ For isolate 2158 which has an MIC value close to the breakpoint concentration for ciprofloxacin, in the higher inoculum sample the resazurin was slowly changed in the presence of antibiotic, whereas at the threshold concentration (*i.e.*, lower inoculum cell density) there was no reduction in colour in the presence of antibiotic (Fig. 3), suggesting that in general lower starting cell densities may improve AST accuracy. At standardised cell dilutions we found identical susceptibility profiles to conventional microplate BMD. To achieve rapid time-to-result without risking false-resistance results at higher starting cell density, the simplest approach may be to test multiple serial dilutions of urine in parallel. The most rapidly detected growth curves can be discounted due to high starting cell density, and susceptibility determined at later timepoints where starting cell density can be assumed to be low enough for accurate results. This may justify the additional sample handling step of serial dilution step, depending on bacterial densities expected to be found in the urine samples to be tested.

These spiked urine samples represent a simple model of urinary tract infection samples. While many uncomplicated infections can be characterised by a single organism at high density, other infections may involve mixed cultures or asymptomatic bacteriuria which is not causing an infection. Mixed samples are more difficult to interpret but could be combined with rapid ID methods, such as MS, and need consideration of patient information, such as age, to interpret and act on test results.

### Direct mcAST in clinical diagnostic remnant urine samples

**Fig. 4:**
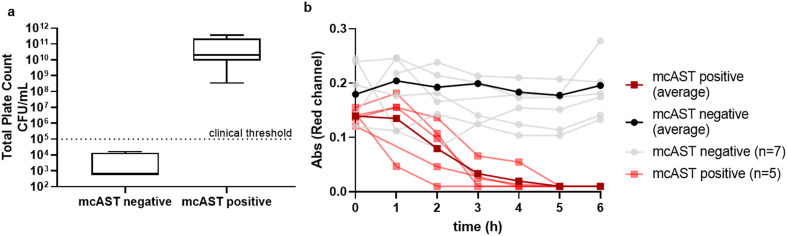
mcAST detection of bacteria positive clinical samples. (a) Total plate count CFU mL^−1^ of 12 urine samples diluted 1 : 100 against positive growth detected without antibiotic. Error bars indicate minimum and maximum, Q2 indicates median. Negative samples marked at LoD of 600 CFU mL^−1^. (b) Resazurin absorbance of 12 clinical samples. Solid colour indicates mean absorbance for positive and negative samples. Semi-transparent colour indicates individual urine samples.

Following this testing of pure cultures spiked into healthy urine, we performed an initial evaluation of mcAST with 12 sequential clinical diagnostic remnant urine samples identified as requiring microbiological investigation, and compared our results with conventional plating and AST. Of these 12 samples, 5 had total plate counts above 10^5^ CFU mL^−1^, the clinical threshold for infection. The diagnostic remnant samples were collected in bacteriostatic boric acid containers used to preserve samples during transportation to laboratory site. We observed that boric acid, even diluted 1 : 5, inhibited bacterial growth, so samples had to be further diluted. When diluted 1 : 100 in resazurin broth, and tested in mcAST, all urines with a negative plate count or with counts below the clinical threshold showed no colour change; whereas plate counts above the clinical threshold all showed resazurin colour change in the absence of antibiotics indicating bacterial growth (Fig. 4a). For detection of bacteria above the clinical threshold, mcAST showed 100% sensitivity and specificity after 6 h incubation. The diagnostic urine samples collected here contained boric acid, a bacteriostatic to preserve urine samples during transportation. The resazurin colour changed was monitored hourly for 6 h. All samples with total plate count above the clinical threshold showed colour change within 6 h, which corresponds with our spiked healthy urine samples (Fig. 4b). This indicates that the urine matrix, at this dilution, does not have a significant effect on growth rate and early time points for AT can be interpreted in clinical samples.

Antibiotic concentrations were chosen around the breakpoint of *Enterobacterales*, the most common pathogen found in UTIs, and the most abundant organism identified in each sample. The direct AST of urine diluted 1 : 100 was compared to the AST of pure cultures of the most abundant bacteria isolated from them (Table 2). Of the 5 positive samples we identified 4 samples with mixed growth and 1 sample with only *E. coli* present. The most abundant organisms identified were *E. coli* or coliform, although mixed samples also contained lower levels of *Pseudomonas* sp. and *Enterococcus* and other coliforms. The direct AST showed good agreement to the reference method of the most abundant organisms, with only one false resistant result identified in urine 3 for cephalexin. The false resistant result had the highest total plate count encountered, which may indicate that a high inoculum is causing false resistance. We also predict the mcAST would be expected to detect the most resistant strain of bacteria present in the urine sample (densities above the LoD of 5 × 10^3^ CFU mL^−1^ after dilution). Further testing of clinical samples is now required to fully establish the accuracy, and identify in more detail the effect of inoculum and AST profiles in mixed growth samples from larger panels of patients.

**Table 2:** Susceptibility results of direct urine AST and the most abundant isolated bacteria after 6 h

Urine ID	Organism ID	TPC^a^ (CFU mL^−1^)	1 : 100 urine dilution				Most abundant isolate ID				
			NITRO	CEFA	CIPRO	TRIM	Organism ID	NITRO	CEFA	CIPRO	TRIM
1	*E. coli*	3.52 × 10^8^	≤64 (S)	≤16 (S)	≤0.25 (S)	≤4 (S)	*E. coli*	≤64 (S)	≤16 (S)	≤0.25 (S)	≤4 (S)
2	Mixed growth	3.61 × 10^11^	≤64 (S)	>32 (R)	≤0.25 (S)	≤4 (S)	*E. coli*	≤64 (S)	≤16 (S)	≤0.25 (S)	≤4 (S)
3	Mixed growth	8.7 × 10^10^	≤64 (S)	≤16 (S)	≤0.25 (S)	>8 (R)	*E. coli*	≤64 (S)	≤16 (S)	≤0.25 (S)	>8 (R)
4	Mixed growth	1.8 × 10^10^	≤64 (S)	≤16 (S)	≤0.25 (S)	≤4 (S)	*Coliform*	≤64 (S)	≤16 (S)	≤0.25 (S)	≤4 (S)
5	Mixed growth	2 × 10^10^	≤64 (S)	>32 (R)	≤0.25 (S)	>8 (R)	*E. coli*	≤64 (S)	32 (R)	≤0.25 (S)	>8 (R)
		Concordance (%)	**100 (5/5)**	**80 (4/5)**	**100 (5/5)**	**100 (5/5)**					

a TPC indicates total plate count. NITRO: nitrofurantoin, CEFA: cefalexin, CIPRO: ciprofloxacin, TRIM: trimethoprim.

### Mass production antibiotic deposition method for scalable manufacture

Although we have previously presented proof-of-concept for mcAST using melt-extruded strips,^42,50^ production of such tests must be scalable to maintain affordability; there is no benefit from the mass-production of the plastic devices if they are slow and expensive to load with assay reagents. An engineering problem is linked to deposition of AST reagents within the microcapillaries. While freeze drying the antibiotics into the capillaries deposits a known concentration of antibiotic, simplifying validation of the test, it is a relatively low-throughput manufacturing technique not compatible with high volume production, as each test strip needs to either be cut to size and then the liquid antibiotics injected; or cut to size after the liquid sample is loaded, which may lead to uneven loading. Furthermore, freeze-drying at scale requires significant capital investment and batch manufacturing.

**Fig. 5:**
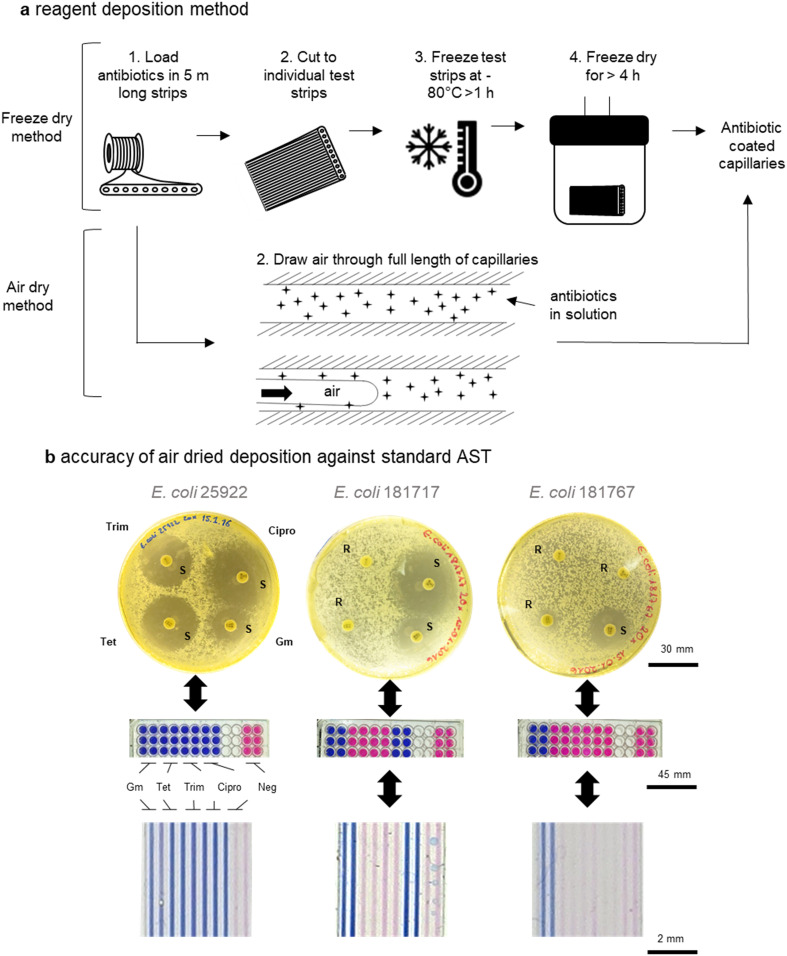
Antibiotic loading methods for mcAST devices and comparison with conventional AST methods. a Low-throughput freeze drying antibiotic deposition method requires more steps to produce capillaries deposited with antibiotic. The simplified mass-manufacture air drying method can be performed on bulk microcapillary lengths. b Comparison of mcAST categorical agreement with conventional disc diffusion and microplate broth microdilution when loaded using mass manufacturing method. Disc diffusion matches microplate method and mcAST results. Set performed on two UPEC isolates and *E. coli* quality control strain 25922.

Many microfluidic systems developed in research laboratories are often produced either manually or using highly specialised equipment, thereby not using affordable or scalable techniques.^20^ Here, we show that air drying of antibiotic reagents is reliable and can be used to increase production of mcAST strips by allowing loading and drying of bulk strips before they are cut to size. As previously, several metres of microcapillary film can be loaded in bulk (representing hundreds of test strips). Multiple lengths of the microcapillary film can then be attached to a vacuum pump *via* a manifold. As the air is drawn through the capillaries a thin film of reagent is left behind.^42^ The lengths of antibiotic coated microcapillary film can then be cut to the desired test size (Fig. 5a). The deposition of reagent can be tuned based on the velocity of reagent removal. The addition of polymers to increase the viscosity of the antibiotic solution can increase reagent deposition therefore the best loading solution can be optimised for increased reagent deposition (Fig. S-4†). The air dried method also requires less costly equipment compared to a freeze drier. Here air drying was performed on a simple vacuum manifold but for further control of the system, this could be performed by pushing clean dry compressed air or nitrogen through the system with a regulator.

To validate the air-drying method, four antibiotics, gentamicin, tetracycline, trimethoprim and ciprofloxacin, were tested. Disc diffusion in agar plates and endpoint resazurin mcAST devices were performed on two clinical *E. coli* UTI isolates plus the quality control strain (ATCC 25922) for comparison with mcAST (Fig. 4b). Loading concentrations were calculated based on previous studies of antibiotic loading efficiency by LC-MS^42^ and functional AST experiments (Fig. S-5†). While the concentration of antibiotic deposited was not fully quantified here, it was clear that the loading of a high concentration of antibiotic, followed by removal, leaves behind a deposit of antibiotic that functionally matches the results obtained in a microplate (Fig. S-5†). We found full agreement between the novel mcAST, microtitre plates and disc diffusion (Fig. 5b). Further stability studies are warranted to establish the shelf life of antibiotic-loaded mcAST test strips.

## Conclusions

This study provides proof of concept design of a point of care AST system. We show that the concept of direct antibiotic susceptibility testing using simulated fresh urine samples and diagnostic remnant urines containing boric acid can determine antibiotic susceptibility within 6 h. Together, using a portable 3D printed PoC–mcAST, the simple operation of the sample loading allows the test to be performed outside a laboratory. The colorimetric readout allows for simple digital results capture with a smartphone camera. While many microfluidic systems struggle with low-cost manufacture and difficulties in reagent deposition, we display two manufacturing methods for incorporating dry antibiotic within test strips that both result in accurate AST profiles.

A major challenge for direct AST is the unknown inoculum density. We showed the time-to-result depends strongly on cell density, nevertheless the kinetics of degradation or conversion of resazurin intrinsically linked to the rate of bacteria growth kinetics closely followed that observed for a macrofluidic microtiter well plate. However, some impact of starting cell density on detection of antibiotic susceptibility was observed, with the highest starting cell densities showing evidence of growth in the presence of some antibiotics, that could lead to false resistance. Further growth measurement with different inoculum densities in the presence of antibiotics may be needed, for example using serial sample dilution, to identify the optimal dilution factor and readout times. We can hypothesise that the maximum wait time for threshold infection of a Gram-negative bacterial infection would be in the order of 6 h, a significant improvement from the >48 h under the current standard clinical laboratory testing pathway. False resistance results can potentially be avoided simply by testing serial dilutions of urine and ignoring dilutions where rapid detection indicates high starting cell density.

## Ethical approval

The collection of urinary pathogenic *E. coli* from a tertiary care hospital of Pakistan from community acquired UTI patients and was approved by Ethical Review Board (ERB) of Pakistan Institute of Medical Sciences. Ethical consent for the collection of urine from healthy donors was received from the University of Reading, reference code 19/59. Informed written consent was obtained from all participants. The collection of diagnostic remnants from Basingstoke and North Hampshire Hospital was approved by the Health Research Authority (HRA) (IRAS: 316558) and the University of Reading, reference code 22/33.

## Author contributions

SHN: data curation, funding, formal analysis, investigation, methodology, project administration, software, visualization, writing – original draft, writing – review & editing. JP: investigation, formal analysis, investigation, methodology, visualization, writing – original draft. ADE: conceptualisation, funding acquisition, methodology, project administration, supervision, writing – original draft, writing – review & editing. SPK: project administration, supervision, funding, writing – review & editing. HL: investigation, writing – review & editing. JH: investigation, methodology, writing – review & editing. NMR: investigation, formal analysis, visualisation, methodology, writing – review & editing. MW: resources, methodology, writing – review & editing. TD: investigation, methodology, writing – review & editing.

## Conflicts of interest

A. D. Edwards and N. M. Reis are the inventors of patent application protecting aspects of the novel microfluidic devices tested in this study and is a director and shareholder in Capillary Film Technology Ltd, a company holding a commercial license to this patent application: WO2016012778 “Capillary assay device with internal hydrophilic coating” inventors AD Edwards, NM Reis.

## Supplementary Material

SD-002-D2SD00138A-s001
