# Supplementary material for: Moving microcapillary antibiotic susceptibility testing (mcAST) towards the clinic: unravelling kinetics of detection of uropathogenic E. coli, mass-manufacturing and usability for detection of urinary tract infections in human urine
Source: Sens Diagn. 2023 May 3;2(3):736–50. doi: 10.1039/d2sd00138a (PMC10197089; doi:10.1039/d2sd00138a)
Supplement: SD-002-D2SD00138A-s001 [file SD-002-D2SD00138A-s001.pdf]

Electronic Supplementary Material (ESI) for Sensors & Diagnostics.
This journal is © The Royal Society of Chemistry 2023

Supporting Information

Moving microcapillary antibiotic susceptibility testing (mcAST) towards the clinic: unravelling kinetics of detection of uropathogenic *E. coli*, mass-manufacturing and usability for detection of urinary tract infections in human urine

Sarah H Needs[a,*], Jeremy Pivetal[a], Jessica Hayward[a], Stephen P Kidd[b], HoYin Lam[b], Tai Diep[a], Kiran Gill[a], Martin Woodward[c], Nuno M Reis[d,e*], Alexander D Edwards[a,e,*]

[a]Reading School of Pharmacy, University of Reading, Whiteknights Campus, Reading, RG6 6AD United Kingdom

[b]Hampshire Hospitals NHS Foundation Trust, Basingstoke and North Hampshire Hospital, Basingstoke, RG24 9NA, UK

[c]Department of Food and Nutrition Sciences, University of Reading, Whiteknights Campus, Reading, RG6 6DX, United Kingdom

[d]Department of Chemical Engineering and Centre for Biosensors, Biodevices and Bioelectronics (C3Bio), University of Bath, Claverton Down, Bath BA2 7AY, United Kingdom

[e]Capillary Film Technology (CFT), Daux Road, Billingshurst, RH14 9SJ, United Kingdom

TABLE OF CONTENTS

SUPPLEMENTARY METHODS pp 2

Microplate Broth Microdilution pp 2

Disc Diffusion pp 3

Portable incubator and imaging system pp 3

Kinetic model for resazurin metabolization pp 3

SUPPORTING TABLES pp 5

SUPPORTING FIGURES pp 6

SUPPLEMENTARY METHODS

Microplate Broth Microdilution

Microplate BMD was performed as described (Needs et al., 2021c), 3-4 colonies per agar plate were inoculated in 1 mL of Mueller-Hinton Broth (MHB) and incubated shaking at 37 °C for several hours. Cultures were diluted to a 0.5 McFarland standard using a spectrophotometer, diluted 1:100 in MHB. Finally 50 µL of culture was added to microplate wells containing 50 µL of antibiotic solution and resazurin at a concentration of 0.5 mg/mL. Plates were incubated overnight at 37 °C.

Disc Diffusion

Clearly isolated single colonies from a freshly streaked (18- to 24-hour incubation) bacterial plate were suspended in a saline solution (0.8% NaCl), adjusted to a 0.5 McFarland, diluted 10-fold in a Mueller-Hinton broth (Sigma Aldrich) and further diluted 10-fold in a Mueller-Hinton broth. Inoculum were then spread onto Mueller-Hinton agar plate and antibiotic impregnated discs (Oxoid, UK) of gentamicin (10 µg), tetracycline (30 µg), trimethoprim (2.5 µg) and ciprofloxacin (1 µg) were deposited and incubated overnight at 37°C.

Portable incubator and imaging system: Bill of Materials - Hardware

Component	Quantity	Cost	Source
TalentCell Rechargeable 36 W 12 V/6000mAh 5 V/12000mAh DC Output Lithium-Ion Battery Pack	1	£59.99	amazon.co.uk
Aideepen STC-1000 DC 12 V-72 V LED Digital Temperature Controller	1	£8.69	amazon.co.uk

RS PRO Red and Black 0.75 mm² Equipment Wire, 18 AWG, 24/0.2 mm, 1m	1	£0.60	RS Components
WAGO Lever 3-way (Part 222–413)	2	£0.60	RS Components
ABS Filament for printing – 1.75mm	1	£23.50	Ooznest
Mini Heated Bed - OpenBuilds	1	£11.50	Ooznest
Mirrored 3mm acrylic 100 x 120 mm	1	£1.58	cutmyplastic.co.uk
Opal diffuse 3 mm acrylic 100 x 120 mm	1	£1.64	cutmyplastic.co.uk
Design files:			
3D printed incubator-reader design file	1	-	https://gitlab.com/sneeds/bacterial_testing/-/blob/master/pASTa_3x3_v3-sample_incubator-reader_120cube__1_.stl
3x3 incubator tray	1	-	https://gitlab.com/sneeds/bacterial_testing/-/blob/master/tray.stl
mcAST clip holders	1	-	https://gitlab.com/sneeds/bacterial_testing/-/blob/master/MCF_CLI_INDIVID.stl

Kinetic model for resazurin metabolization

We have modelled the rate of metabolization of resazurin as associated to the cell density as follows. In the presence of unlimited substrate concentration and small cell density or CFU/ml, the rate of increase in bacteria density C_X follows a standard exponential growth described by:

$$\frac{dC_X}{dt} = \mu \cdot C_X \tag{1}$$

where μ is the growth rate of the bacteria (hr^{-1}). On the other hand, metabolization of resazurin has been shown to be a biocatalytic process, with the rate of metabolization of resazurin being proportional to the biomass concentration or cell density, C_X (in units of CFU). Assuming the rate of metabolization of resazurin dC_R/dt is independent to the concentration of resazurin, C_R, this can be represented a first order reaction:

$$\frac{dC_R}{dt} = k_c C_X \qquad (2)$$

where k_C is a kinetic constant representing the number of moles of resazurin converted per unit of time per CFU (mol CFU^{-1} hr^{-1}).

Integration of Eq. (1) allows estimating the cell density, C_x over time, t during exponential growth, considering the initial cell density in the inoculum, $C_{X,0}$:

$$C_X(t) = C_{X,0} exp^{(\mu\, t)} \qquad (3)$$

Combining Eqs (2) and (3), yields:

$$\frac{dC_R}{dt} = k_c C_{X,0} exp^{(\mu\, t)} \qquad (3)$$

Conversion, X of resazurin can be defined as:

$$X = \frac{C_{R,0} - C_R}{C_{R,0}} \qquad (4)$$

Where $C_{R,0}$ is the initial concentration of resazurin and C_R the resazurin concentration at a given time t. Note X varies between 0 (t = 0) and 1 (full conversion). The initial concentration of resazurin in the experiments is known (described in materials and methods). C_R is not measured along the incubation time however it can be shown that concentration of resazurin is linearly proportional to the Abs/cm measured throughout the experiments, especially in the microcapillaries due to the very short light patch distance, in line with Lambert-Beer law.

Integrating Eq. (3) with boundary conditions $C_{R,0}$ for t = 0 and C_R for t = t and taking into account definition of X as described in Eq. (4), yields:

$$X = \frac{k_c\, C_{x,0}}{\mu\, C_{R,0}}\left[exp^{(\mu\, t)} - 1\right] \qquad (5)$$

Which is able to predict the degree of metabolization or conversion of resazurin during incubation time. With the growth rate μ being estimated from experimental data, the kinetic model in Eq. (5) yields a single unknown kinetic constant k_c (mol CFU^{-1} hr^{-1}), which can be found by best-fitting the model to the experimental data using Excel's solver using as criteria minimum square differences. Note that for simplicity often the term (1-X) *vs* time has been selected in order to match the dynamics of the experimental data, with absorbance linked to the amount of unconverted resazurin in the growing media.

SUPPORTING TABLES

Table S-1. Breakpoint concentrations for Enterobacterales from EUCAST Breakpoint Tables V 11.0

**Antibiotic**	**Breakpoint Concentration (mg/L)**	**ATU**	**Antibiotic Concentration tested (mg/L)**
Nitrofurantoin	≤64	-	64
Cefalexin	≤16	-	16
Ciprofloxacin	≤0.25	0.5	0.25
Trimethoprim	≤4	-	4
Cefoxitin	≤8	-	8 - 16
Amikacin	≤8	-	8 – 16

Table S-2. Time for resazurin conversion in 11 different urine samples diluted to 90%, 50% and 20% urine in Mueller-Hinton broth.

**Urine Code**	**Time to resazurin conversion (h)**		
	**90% urine**	**50% urine**	**20% urine**
Mueller-Hinton Broth	1.93		
007-1219-01	2.41	2.11	2.13
001-1219-02	2.67	2.30	2.23
006-1219-01	-	2.19	1.93
020-1219-01	-	-	2.48
013-1219-01	-	2.92	2.61
001-1219-03	-	1.44	2.14
009-1219-01	3.96	3.12	2.48
014-1219-01	2.31	2.51	2.60
012-1219-01	3.25	2.50	2.37
001-1119-01	-	1.21	2.10
004-1119-01	-	-	2.04

Urine was diluted to 20% final concentration and tests were monitored every 15 minutes for 6 h. The time to resazurin conversion based on the threshold concentration diluted 1:5 in MHB is 4.9 h (95% CI 4.7-5.2 h) (Needs et al. 2021), therefore an endpoint of 6 h was used for all tests. There was 100% categorical across nitrofurantoin, cefalexin and trimethoprim for 20 UPEC isolates and E. coli 25922,

spiked into two different urine samples using two starting inoculum densities, compared to gold standard microplate broth microdilution (Table 2). Ciprofloxacin showed 95% agreement at the high bacterial load corresponding to $3x10^7$ CFU/mL in urine, above the density recommended for AST measurements. Even at the clinical threshold of 10^5 CFU/mL there was 90% categorical agreement for ciprofloxacin. This issue is mostly due to the MIC of some of the UPEC isolates being within ± 1 doubling dilution of antibiotic. This would be a problem even when using that gold standard technique as MIC varies within labs.

SUPPORTING FIGURES

The portable hotbed stays with ± 2 degrees of the temperature set on the microcontroller.

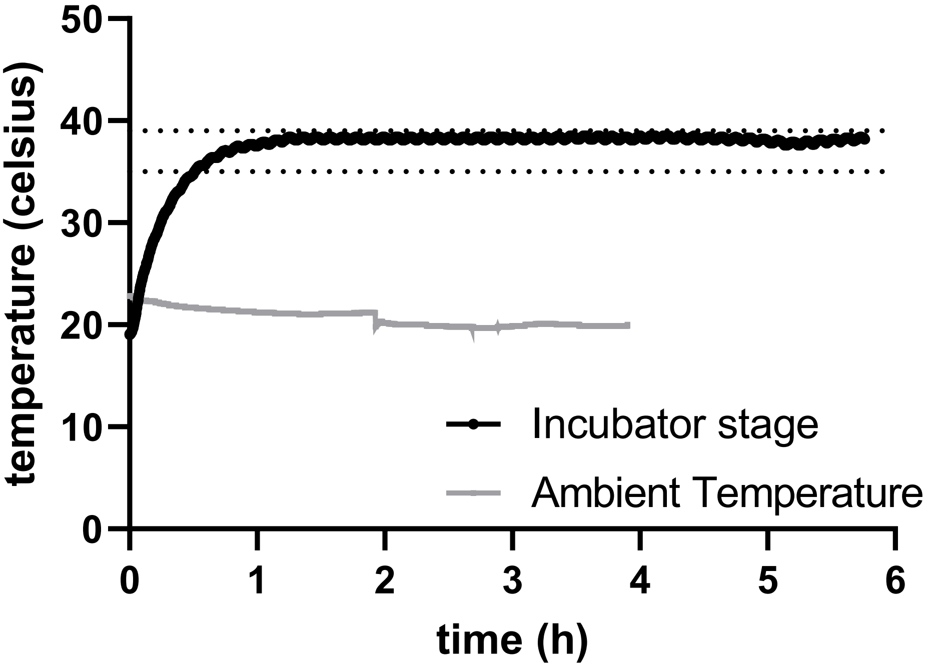

Figure S-1. Hotbed set to 37 degrees Celsius and run on battery power for 6 h. Dotted lines indicate 35 and 39 degrees Celsius.

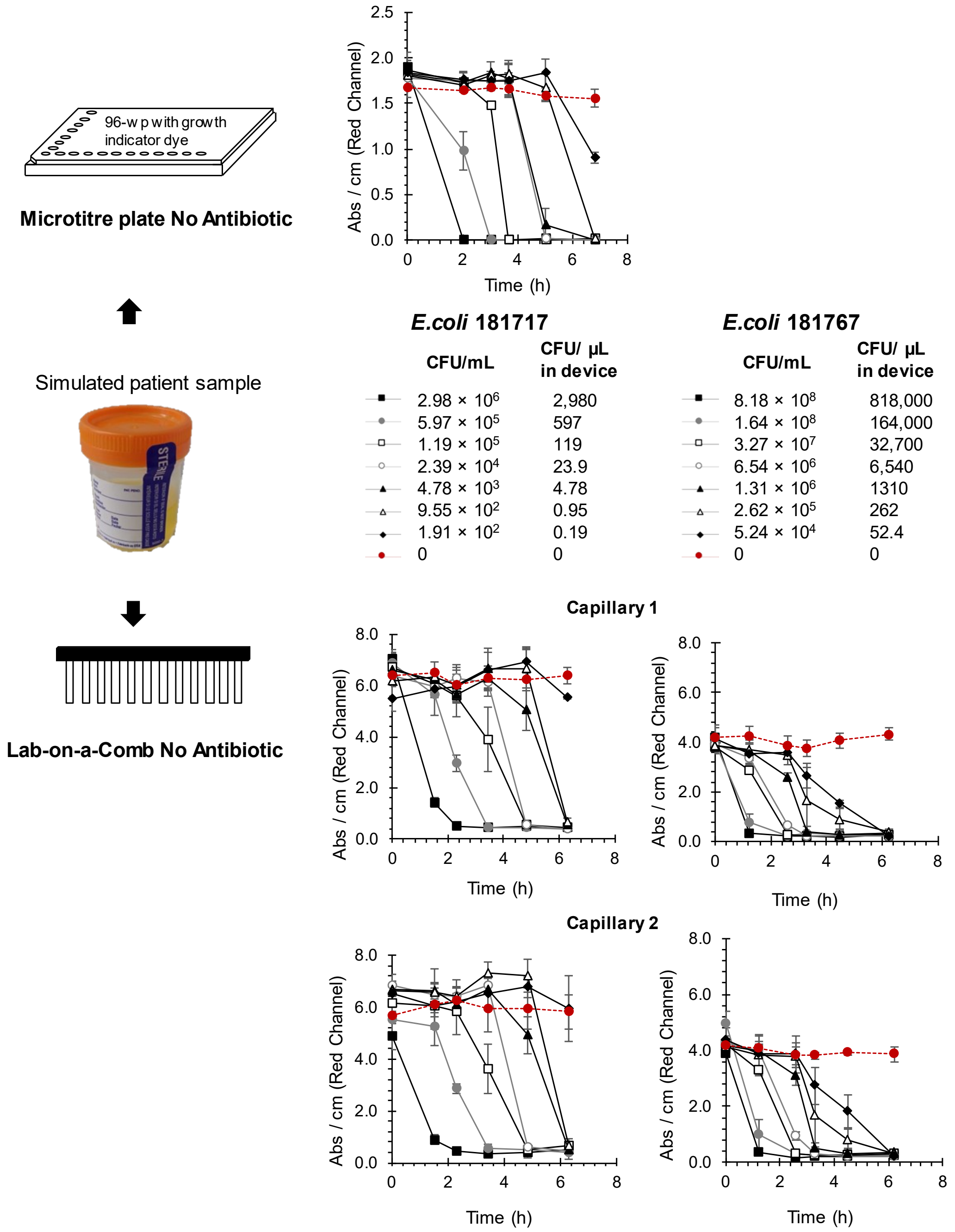

Figure S-2. Replicates of detection kinetics from simulated clinical urine samples between microcapillaries at clinically relevant range of starting pathogen density.

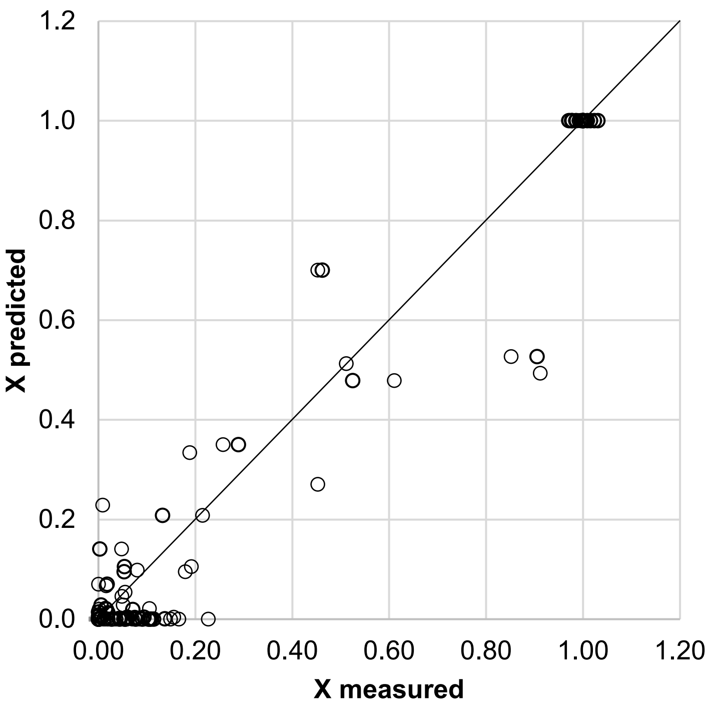

Figure S-3. Comparison of experimental resazurin conversion in the microtiter plate and microcapillaries vs predicted conversion from the kinetic model.

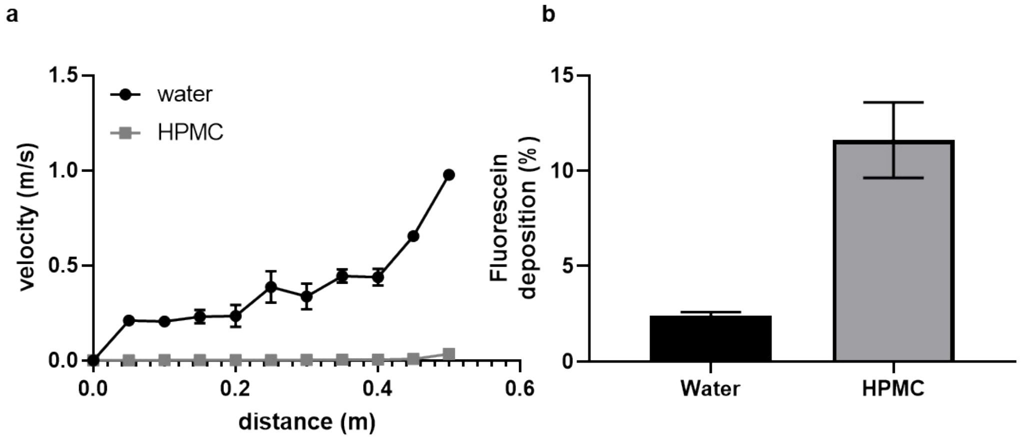

Figure S-4. Velocity changes reagent deposition. Fluorescein was diluted in water or 10 mg/mL HPMC. **a** Velocity was calculated every 10 cm by filling a 1m length of MCF with the test liquid and videoing the removal when attached to a vacuum pump and left on the pump for 20 minutes. **b** The 1m lengths were cut into 10 cm and dipped into water until filled and imaged under blue LEDs. The average fluorescent intensity was calculated for each strip and compared to a fluorescein calibration curve to identify concentration of fluorescein remaining. Data indicates the mean fluorescein concentration ± SD for 10 strips.

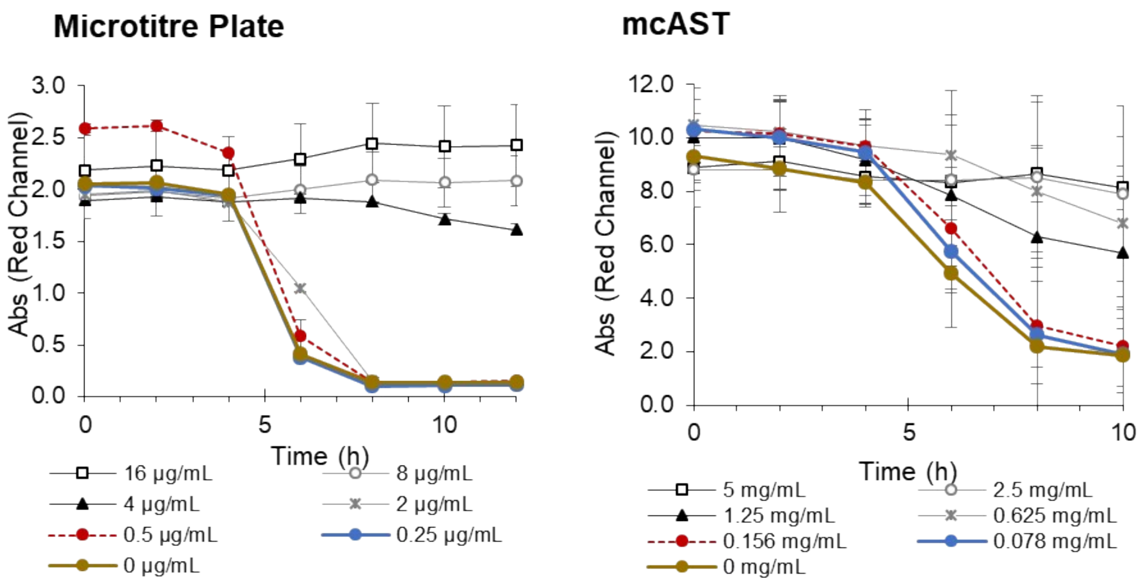

Figure S-5. Comparison of time resolved ampicillin broth microdilution of *E. coli* 25922 performed in microtitre plate and mcAST with antibiotics deposited by air-drying. Antibiotics were loaded at higher concentrations in the mcAST –air dried but functional MIC reports the same result indicating sir dried antibiotics, leaving behind a thin film of antibiotic in the capillaries can be used to accurately measure antibiotic susceptibility.
